# Involvement of Mitochondrial Dysfunction in *FOXG1* Syndrome

**DOI:** 10.3390/genes14020246

**Published:** 2023-01-17

**Authors:** Victoria A. Bjerregaard, Amanda M. Levy, Mille S. Batz, Ravina Salehi, Mathis Hildonen, Trine B. Hammer, Rikke S. Møller, Claus Desler, Zeynep Tümer

**Affiliations:** 1 Kennedy Center, Department of Clinical Genetics, Copenhagen University Hospital, 2600 Glostrup, Denmark; 2 Danish Epilepsy Centre, Department of Epilepsy Genetics and Personalized Medicine, 4293 Dianalund, Denmark; 3 Department of Regional Health Research, University of Southern Denmark, 5230 Odense, Denmark; 4 Department of Cellular and Molecular Medicine, Center for Healthy Aging, University of Copenhagen, 2200 Copenhagen, Denmark; 5 Department of Clinical Medicine, Faculty of Health and Medical Sciences, University of Copenhagen, 2200 Copenhagen, Denmark

**Keywords:** *FOXG1* syndrome, neurodevelopmental disorders, mitochondrial dysfunction, mitochondrial homeostasis, mitochondrial morphology, mitochondrial respiratory capacity

## Abstract

*FOXG1* (Forkhead box g1) syndrome is a neurodevelopmental disorder caused by a defective transcription factor, FOXG1, important for normal brain development and function. As *FOXG1* syndrome and mitochondrial disorders have shared symptoms and FOXG1 regulates mitochondrial function, we investigated whether defective FOXG1 leads to mitochondrial dysfunction in five individuals with *FOXG1* variants compared to controls (*n* = 6). We observed a significant decrease in mitochondrial content and adenosine triphosphate (ATP) levels and morphological changes in mitochondrial network in the fibroblasts of affected individuals, indicating involvement of mitochondrial dysfunction in *FOXG1* syndrome pathogenesis. Further investigations are warranted to elucidate how FOXG1 deficiency impairs mitochondrial homeostasis.

## 1. Introduction

*FOXG1* (Forkhead box g1) syndrome (OMIM #613454) is a neurodevelopmental disorder caused by heterozygous pathogenic variants in *FOXG1*, encoding FOXG1. Individuals with loss-of-function (LoF) variants (larger deletions or intragenic sequence variants) and duplications present with different symptoms [1]. The clinical features associated with LoF variants are generally more severe and include global developmental delay, microcephaly, dyskinetic–hyperkinetic movement disorders, stereotypies, speech and visual impairment, breathing abnormalities, sleep disturbances, and epilepsy, while individuals with duplications are less severely affected [1].

FOXG1 is an evolutionarily highly conserved nuclear–cytosolic transcription factor that plays an essential pleitropic role in arealization, lamination, and neurogenesis in the developing brain [1]. In the developing mouse brain, Foxg1 functions as a transcriptional repressor in the nucleus, preventing neuronal progenitor cells from undergoing premature differentiation, and in the cytoplasm, it promotes neuronal differentiation [2]. Comprehensive studies on *Foxg1* heterozygous mice have described various features similar to the symptoms observed in *FOXG1* syndrome [3]. Haploinsufficiency of *Foxg1* in mice and *FOXG1* in human induced pluripotent stem cell (iPSC)-derived neurons and organoids alters the balance of excitatory and inhibitory synaptic protein expression leading to a decrease in brain synapses and may contribute to the neurodevelopmental phenotype and explain early-onset seizures which are characteristic of the disorder [4,5,6]. A fraction of Foxg1 has more recently been shown to localize to the mitochondria in rodents and coordinates cell differentiation, mitochondrial dynamics, and bioenergetics [7]. Notably, the mitochondrial dynamics of the cell appears to be determined by an interplay between nuclear and mitochondrial Foxg1, where the nuclear protein enhances the mitochondrial membrane potential (MMP) and induces mitochondrial fission, while the mitochondrial protein promotes mitochondrial fusion events [7].

FOXG1 has three main functional domains: FBD (Forkhead DNA binding domain, amino acids 181–275), GBD (Grouch binding domain, amino acids 307–317), and JBD (JARID1B domain, amino acids 383–406). The FBD harbors nearly all the pathogenic *FOXG1* missense variants [1], which are likely to have an LoF effect. Phosphorylation of Ser19 at the N-terminal promotes nuclear import of Foxg1, while phosphorylation of Thr279 controls cytosolic localization [2]. Amino acids 285–309 (mouse 277–301) were recently shown to be essential for the mitochondrial localization of the protein [7].

Mitochondria are highly dynamic organelles that rapidly adapt to the variable energy demands of the cell by changing their abundancy, morphology, and subcellular distribution through continuous fission and fusion events [8]. Through oxidative phosphorylation, mitochondria provide most of the energy required by human cells in the form of adenosine triphosphate (ATP). Deregulation of mitochondrial dynamics and homeostasis is linked to energy deprivation which can cause a broad range of clinical manifestations, especially neurological symptoms including epilepsy and movement disorders, which are also characteristic for *FOXG1* syndrome [9]. However, the contribution of mitochondrial dysfunction to the pathogenesis of *FOXG1* syndrome has not yet been investigated.

All these aspects, together with the suggested role of FOXG1 in mitochondrial function, lead us to hypothesize that mitochondrial dysfunction plays a role in the pathogenesis of *FOXG1* syndrome. To address this, we investigated *FOXG1* expression and mitochondrial function in skin fibroblasts from five affected individuals with different intragenic *FOXG1* variants and six healthy controls. To our knowledge, this is the first study to suggest involvement of impaired mitochondrial homeostasis and reduced respiratory capacity in the pathogenesis of *FOXG1* syndrome.

## 2. Materials and Methods

### 2.1. Fibroblasts and Cell Culturing

Skin fibroblasts from five individuals with *FOXG1* syndrome (mean age 15.2 years) and six healthy individuals (mean age 9.8 years) were included in the study (Supplementary Table S1). The fibroblasts were obtained from the Coriell Biorepository or from affected individuals followed at the Danish Epilepsy Center, Dianalund. Three of the variants were missense residing in the FBD domain (p.(Leu189Phe), P5; p.(Phe215Val), P4; and p.(Arg230His), P3). Of the two truncating variants, one was a nonsense within the GDB domain (p.(Trp308*), P1), and the other was a single nucleotide duplication at the N-terminal of the protein (p.(Gln86Profs*35), P2). All the variants were previously reported in affected individuals (HGMD database) and absent in control populations (gnomAD database, v2.1.1) The variants are described according to NM_005249.5. The fibroblasts were cultured in Dulbecco’s modified eagle medium (DMEM) (Gibco, Waltham, MA, USA) supplemented with 10% FBS (Gibco) and 1% penicillin/streptomycin, at 37 °C in a humidified atmosphere with 5% CO2. Cells were routinely tested for mycoplasma. Fibroblasts obtained from individuals with *FOXG1* variants are defined as FOXG1-fibroblasts throughout the text.

### 2.2. Mitochondrial Morphology

Mitochondria were stained with COX-IV antibody ([10]; 1:400, ab16056, Abcam, Cambridge, UK) and secondary antibody Goat Anti-Rabbit IgG H&L Alexa Fluor^®^ 488 (Abcam) and imaged on a Zeiss LSM700 confocal microscope. Image segmentation was performed by machine learning using the ZEN Intellesis software (Zeiss, Oberkochen, Germany). Mitochondrial count and network analysis were performed by skeletonization and skeleton analysis of segmented images using ImageJ (v1.53, National Institutes of Health, Bethesda, MD, USA). The mitochondrial shape of the individual mitochondria was measured using the “Shape Descriptor” plugin for ImageJ and extraction of the aspect ratio (AR) (major axis/minor axis).

### 2.3. Mitochondrial Mass

The mitochondrial mass of active mitochondria was quantified by staining with MitoTracker Green (Molecular Probes, Invitrogen, Waltham, MA, USA) and detection by high-throughput microscopy (Nucleocounter 3000, ChemoMetec, Allerod, Denmark). Briefly, cells were incubated with 100 nM MitoTracker for 20 min at 37 °C and washed once in PBS prior to harvest. Cells were then resuspended in 10 ug/mL Hoechst 33,342 (Tocris, Bristol, UK), incubated 10 min at 37 °C, and immediately analyzed. Only live cells were included in the analysis, and the mean fluorescence intensity (MFI) was based on a minimum of 5000 cells. For each sample, a negative control (without MitoTracker) was subtracted from the MFI.

### 2.4. Gene Expression Analysis

RNA was extracted from fibroblasts following standard procedures. Total RNA (1 µg) was used for cDNA synthesis using the High-Capacity cDNA Reverse Transcription Kit (Applied Biosystems, Waltham, MA, USA) according to the manufacturer’s protocol; mRNA expression of *FOXG1* in fibroblasts was investigated using reverse transcription PCR (RT-PCR) using the HotStarTaq^®^ DNA Polymerase kit (Qiagen, Hilden, Germany) followed by 2% agarose gel electrophoresis (Sigma Aldrich, St. Louis, MO, USA). RT-PCR products were verified by Sanger sequencing using the BigDye™ Terminator v3.1 Cycle Sequencing kit and an ABI 3730 DNA analyzer (Applied Biosystems). Quantitative PCR (RT-qPCR) was performed using TaqMan probes from Applied Biosystems. Samples were amplified in triplicates on a 7500 Fast Real-Time PCR system (Applied Biosystems). The relative standard curve method was used for the calculation of mRNA expression levels, and the values of the genes of interest were normalized to the values of *GUSB* or *GAPDH*. All primers, probes, and conditions are listed in Supplementary Tables S2 and S3.

### 2.5. Protein Quantification

Protein levels were quantified by western blot analysis. Briefly, protein was extracted from the fibroblasts using cell extraction buffer (FNN0011, Life technologies), supplemented with Protease Inhibitor Cocktail (Roche, Basel, Switzerland). For SDS-PAGE, whole-cell lysates equivalent to 30 µg of protein were incubated for 10 min at 70 °C with NUPAGE SDS sample buffer (Thermo Fisher Scientific, Waltham, MA, USA) and run on 4–12% or 4–20% Novex Tris-Glycine gels. Proteins were transferred to a Hybond-PVDF membrane (Thermo Fisher Scientific), followed by membrane blocking in 1xPBS- 0,1% TritonX-100 with 5% non-fat dry milk (Sigma Aldrich for 1h at RT. The membrane was then incubated overnight with primary antibodies at 4 °C and with secondary antibodies for 2h at RT. Pierce™ ECL western blotting Substrate (Thermo Scientific) was used for signal detection, and images were captured on a Syngene G:BOX Chemi XRQ chemiluminescence meter. Primary antibodies used were: SOD2 (1:1000, D9V9C; Cell Signaling), LAMP1 (1:200, H4A3, ab25630; Abcam), LC3B-II (1:2000, NB600-1384SS, Novus Biologicals, Englewood, CO, USA), and GAPDH (1:2000, MA5-15738; Invitrogen). Secondary antibodies used were anti-rabbit IgG (1:1000, G-21234; Invitrogen) and anti-mouse IgG, HRP (1:1000, G-21040; Invitrogen).

### 2.6. Mitochondrial Membrane Potential

The MMP was determined by detecting TMRE (tetramethylrhodamine, ethyl ester) (Abcam) by high-throughput microscopy (Nucleocounter 3000). Fibroblasts were incubated with 100 nm TMRE for 15 min at 37 °C, briefly washed in PBS and harvested by standard procedures. Cells were resuspended in 10 µg/mL Hoechst 33,342 (Tocris), incubated for 10 min at 37 °C, and immediately analyzed. A minimum of 5000 live cells were included in the MFI, and 20 µM FCCP (carbonyl cyanide 4-(trifluoromethoxy) phenylhydrazone) (Abcam) was used as a positive control. For each sample a negative control (without TMRE) was subtracted from the MFI.

### 2.7. Superoxide Generation Assay

Mitochondrial reactive oxygen species (ROS) was quantified by MitoSOX red staining (Molecular Probes, Invitrogen) and detection by high-throughput microscopy (Nucleocounter 3000). Fibroblasts were incubated with 5 µM MitoSOX for 20 min at 37 °C followed by a washing step in PBS. Cells were then harvested and incubated for 10 min at 37 °C in 10 µg/mL Hoechst 33,342 (Tocris) and immediately analyzed. The MFI was calculated from a minimum of 5000 live cells, and for each sample, an unstained control (not stained with MitoSOX) was subtracted from the total MFI. Antimycin A (150 µM) (Sigma-Aldrich) was included as a positive control for each experiment.

### 2.8. ATP Content

The ATP content was determined using the luciferase-based assay Vialight MDA Plus kit (Lonza, Basel, Switzerland) according to manufacturer’s instructions. Luminescence was quantified on a Microbeta2 scintillation counter (PerkinElmer, Waltham, MA, USA).

### 2.9. Statistics

An unpaired Student’s t-test was used to compare the means of the FOXG1-fibroblasts to the means of the control fibroblasts obtained from at least three biological replicas; *p*-values below 0.05 were considered significant. Statistical analyses were carried out using Prism software (v 9.0.1, GraphPad, San Diego, CA, USA).

## 3. Results

**Figure 1:**
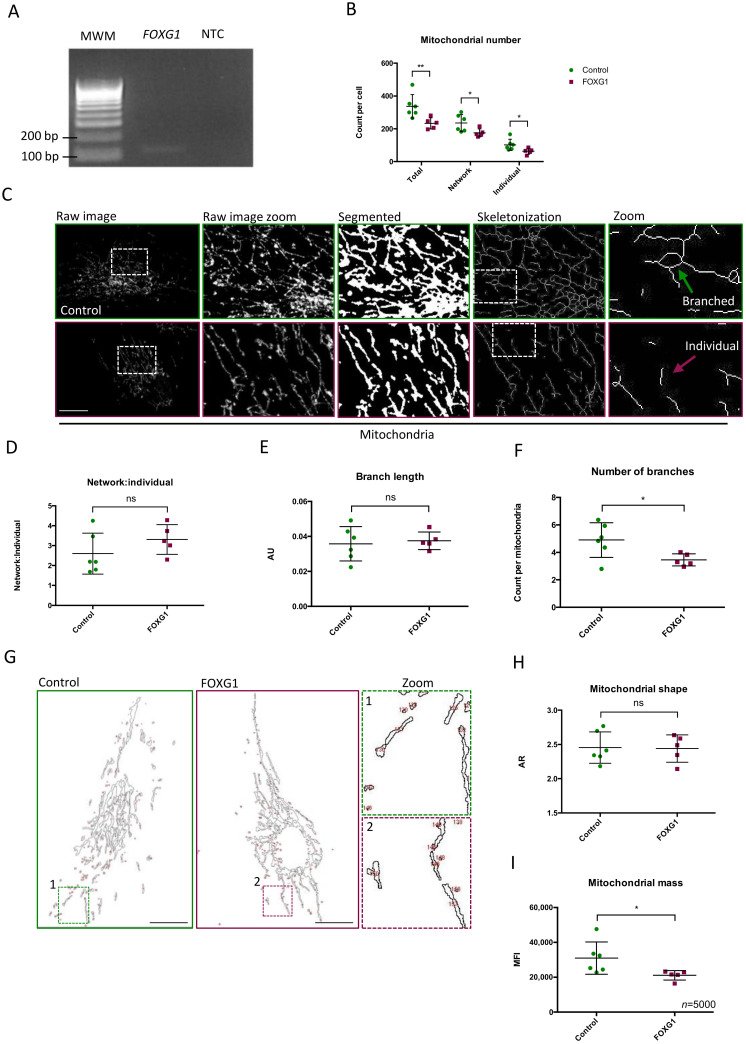
Mitochondrial morphology and content: (**A**) amplification of fibroblast-derived *FOXG1* (Forkhead box g1) cDNA (band size 113 bp); (**B**) mean ± SD mitochondrial number represented as counts per cell of total mitochondria, network mitochondria, and individual mitochondria; (**C**) representative images of mitochondria visualized by COX-IV immunofluorescence and image processed by segmentation and skeletonization; the green arrow indicates branched/network mitochondria; the purple arrow indicates individual mitochondria; (**D**) mean ± SD of ratio between network and individual; (**E**) mean ± SD of branch number per network mitochondria; (**F**) mean ± SD length of network branches represented as arbitrary units (AU); (**G**) representative images of mitochondrial outline to measure the aspect ratio (AR) (major axis/minor axis); (**H**) quantification of the mitochondrial shape presented as mean ± SD AR of mitochondria; (**I**) mitochondrial mass presented as MitoTracker MFI (mean fluorescence intensity) ± SD. Squares and dots represent the mean values ± SD of three biological replicas of each FOXG1- and control subject, respectively; *n* = 20 per biological replica for experiments visualized in Figure B-H; MWM, molecular weight marker; NTC, no template control; SD, standard deviation; *, *p*-value < 0.05; **, *p*-value < 0.01; scale bars = 10 μm.

In this study, we employed skin fibroblasts obtained from five patients with *FOXG1* syndrome (FOXG1-fibroblasts) and six controls. As *FOXG1* is mainly expressed in the brain (Genotype-Tissue Expression (GTEx) portal), we investigated whether *FOXG1* mRNA was expressed in cultured fibroblasts using RT-PCR and verified its expression prior to assessing whether mitochondrial homeostasis was altered in *FOXG1* syndrome individuals (Figure 1A).

### 3.1. Mitochondrial Number, Mass, and Branches Are Decreased in FOXG1-Fibroblasts

To investigate the morphological differences of mitochondria between FOXG1-fibroblasts and controls, individual and networked mitochondria were counted; and the complexity, branch length, and shape of the network were determined by confocal microscopy of COX-IV stained mitochondria (Figure 1). The total number of mitochondria (individual and networked) in FOXG1-fibroblasts was significantly decreased by 30% compared to control fibroblasts (*p* = 0.016) (Figure 1B–C). The ratio between individual and networked mitochondria was unchanged between FOXG1- and control fibroblasts (Figure 1D). The branch length was comparable between the two groups (Figure 1E), while FOXG1-fibroblasts had 29% fewer branches per network (*p* = 0.040) (Figure 1F). We measured the mitochondrial shape expressed as the aspect ratio (major axis/minor axis) but did not observe a difference in the elongation/circularity of individual mitochondria between the two groups (Figure 1G–H).

To verify the decreased number of mitochondria in FOXG1-fibroblasts, mitochondrial mass was determined using MitoTracker Green fluorophore, which accumulates in active mitochondria regardless of MMP. Mitochondrial mass was significantly reduced by 32% in FOXG1-fibroblasts compared to controls (*p* = 0.048) (Figure 1I). The low number of mitochondria together with the low mitochondrial mass indicates a significantly lower mitochondrial content in FOXG1-fibroblasts.

### 3.2. Fission, Fusion, and Mitophagy Do Not Differ between FOXG1- and Control Fibroblasts

**Figure 2:**
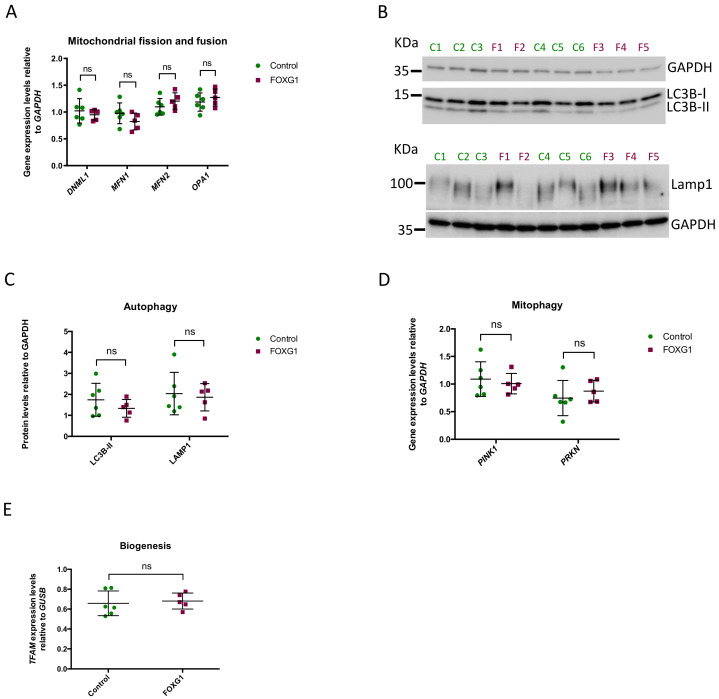
Mitochondrial biogenesis, mitophagy, and fission/fusion: (**A**) mean ± SD of fission protein DRP1 (encoded by *DNM1L*) and fusion proteins *MFN1, MFN2*, and *OPA1* expression levels relative to *GAPDH*; (**B**) representative western blots of the autophagy protein LC3B-II and the lysosomal protein LAMP1 with GAPDH as loading control; (**C**) quantification of western blots normalized to GAPDH; (**D**) mean ± SD of *PINK1* and *PRKN* expression levels relative to *GAPDH*; (**E**) mean ± SD *TFAM* expression levels relative to *GUSB*. Each bar represents mean ± SD of FOXG1- and control fibroblasts obtained from at least three independent experiments. Each square and dot represent the mean values from the experimental replicas for each FOXG1- and control subject, respectively; SD, standard deviation.

As the lower mitochondrial content of FOXG1-fibroblasts could be the result of altered mitochondrial biogenesis, we investigated fission, fusion, and mitophagy. To assess mitochondrial fission and fusion, we quantified the mRNA expression levels of *MFN1, MFN2, OPA1*, and *DNM1L*. In mammalian cells, DRP1 (encoded by *DNM1L*) orchestrates mitochondrial fission, while the fusion of the inner and outer mitochondrial membranes is mediated by OPA1 and MFN1/MFN2, respectively [8]. The mRNA expression levels of all four genes were comparable between FOXG1- and control fibroblasts (Figure 2A). For the assessment of mitophagy levels, we first evaluated the level of general autophagy, quantifying the protein levels of the autophagosome marker LC3B-II and the lysosomal marker LAMP1, which represent the abundance of autophagosomes and lysosomes, respectively (Figure 2B-C, Supplementary Figure S1) [11,12]. We did not observe significantly different levels of either LC3B-II or LAMP1 in the two groups of fibroblasts. Next, we evaluated whether there could be an increase in the recruitment of the autophagy machinery to the damaged mitochondria. This is a process known as mitochondrial priming, where PINK1 activity causes Parkin (encoded by *PRKN*) to bind to the damaged mitochondria to induce their degradation by autophagy [13,14]. We did not observe any difference in the mRNA expression levels of *PINK1* or *PRKN* between FOXG1-fibroblasts and controls (Figure 2D).

These results do not suggest any differences in fission, fusion, or mitophagy between FOXG1- and control fibroblasts, which is substantiated by the observed lack of difference in *TFAM* expression (Figure 2E). The mitochondrial transcription factor TFAM plays a central role in mitochondrial biogenesis as the final modulator of mitochondrial DNA replication and transcription [15].

### 3.3. FOXG1-Fibroblasts Have the Same MMP as the Controls, but Lower Superoxide Production

**Figure 3:**
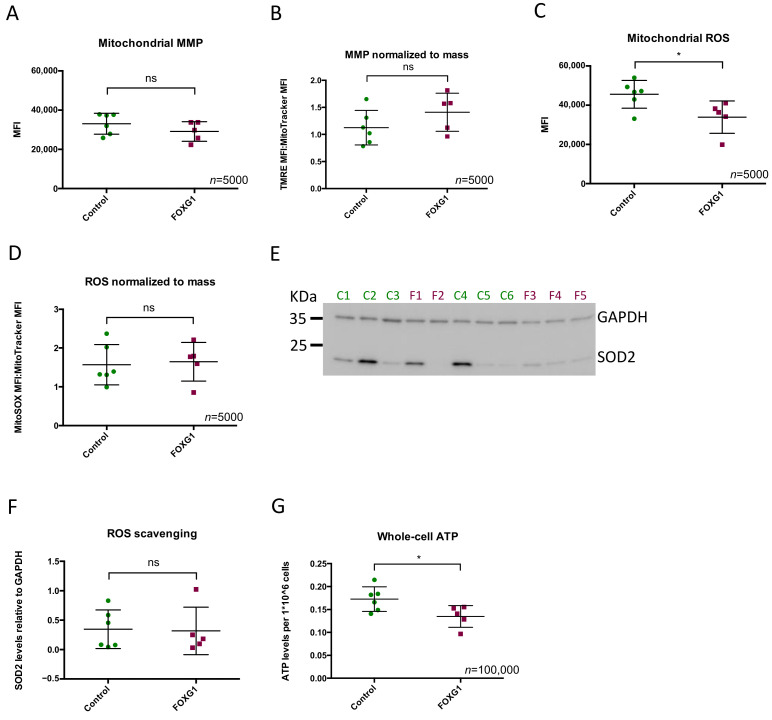
Mitochondrial content and respiratory capacity: (**A**) MMP presented as TMRE (tetramethylrhodamine, ethyl ester) MFI ± SD; (**B**) TMRE MFI normalized to Mitotracker MFI ± SD; (**C**) mitochondrial reactive oxygen species (ROS) represented as MitoSOX MFI ± SD; (**D**) MitoSOX MFI normalized to Mitotracker MFI ± SD; (**E**) representative western blot of the ROS scavenger SOD2 with GAPDH as loading control; (**F**) quantification of western blots normalized to GAPDH; (**G**) whole-cell ATP (adenosine triphosphate) levels represented as mean ± SD ATP levels per 10 × 10^6^ cells. Squares and dots represent the mean values ± SD of at least three biological replicas from each FOXG1- and control subject, respectively; n, number of cells analyzed per biological replica; SD, standard deviation; * *p*-value < 0.05.

We investigated whether the lower content of mitochondria influenced polarization of the mitochondrial membrane using the TMRE fluorophore which labels mitochondria proportional to the potential across the inner mitochondrial membrane [16]. We did not observe any difference in MMP between FOXG1- and control fibroblasts (Figure 3A,B). To assess whether altered mitochondrial dynamics affect mitochondrial superoxide generation, we measured mitochondrial superoxide levels using the mitoSOX Red superoxide indicator. Compared to controls, FOXG1-fibroblasts had significantly lower levels (26%) of superoxide production per cell (*p* = 0.033) (Figure 3C) This difference was leveled out when normalized to the mitochondrial mass (Figure 3D), indicating that the superoxide production per mitochondrion was comparable in FOXG1- and control fibroblasts. As the lower amount of superoxide could be a result of increased ROS scavenging, we measured the level of mitochondrial superoxide dismutase 2 (SOD2). We observed a great variation in SOD2 expression among individual FOXG1- and control fibroblasts (Figure 3E) but no significant differences between the two groups (Figure 3F).

### 3.4. FOXG1-Fibroblasts Have Lower Whole-Cell ATP Levels

To investigate the role of the reduced mitochondrial content on the total bioenergetic of the fibroblasts, we measured whole-cell ATP content (Figure 3G). Whole-cell ATP was significantly lower (22%) in FOXG1-fibroblasts compared to controls (*p* = 0.037) (Figure 3G), which indicates a reduced energetic capacity.

## 4. Discussion

In this study, we investigated involvement of mitochondrial dysfunction in the pathogenesis of *FOXG1* syndrome using fibroblasts obtained from five affected and six healthy individuals. Of the five *FOXG1* variants, two were protein-truncating (one nonsense and one single-nucleotide duplication), and three were missense variants located in the FBD, which is N-terminal to the sequence essential for the mitochondrial localization of the protein [7]. As all the patients were equally severely affected, suggesting a LoF mechanism in each case, and the cohort size was small (*n* = 5), we did not carry out inter-individual assessments but analyzed the combined data of the patient group compared to controls.

For the first time in a human cell model, we have demonstrated that the mitochondrial content and number of network branches are significantly reduced in FOXG1-fibroblasts. In rodents, Foxg1 has been associated with mitochondrial dynamics and bioenergetics [7]. Overexpression of mitochondrial Foxg1 enhances mitochondrial fusion [7], and our observation of a reduced number of branches in mitochondrial networks suggests defective mitochondrial fusion in humans. Although, we did not observe a change in mRNA expression levels of the fusion (*MFN1, MFN2*, and *OPA1*) or the fission (*DNM1L*) genes in FOXG1-fibroblasts compared to controls, posttranslational modification of the factors controlling fusion cannot be ruled out [17].

Notably, while we observed a 30% decrease in mitochondrial content, we did not measure any changes in the MMP in FOXG1-fibroblasts compared to controls. This indicates that although the total number of mitochondria in FOXG1-fibroblasts is decreased, each mitochondrion retains the same membrane potential as control fibroblasts. In contrast, the mitochondrial superoxide levels are decreased in FOXG1-fibroblasts. A decrease in mitochondrial ROS is generally associated with less endogenous damage. However, mitochondrial ROS also serve as signaling molecules that are important for maintenance of cell homeostasis [18], as well as morphogenesis, cell differentiation, and nerve- and skeletal muscle function [19]. From the current data, it is not possible to comment on the putative role of reduced superoxide levels in the neuropathology of *FOXG1* syndrome, and this must be addressed in future studies.

Despite having the same MMP as the control fibroblasts, FOXG1-fibroblasts have a 22% lower whole-cell ATP level. The lower ATP content correlates with a lower mitochondrial content, but to determine the causality of this relationship, further studies are warranted. The low ATP content may be due to other factors that were not investigated in the present study. The contribution from glycolysis could be decreased in FOXG1-fibroblasts, and/or the fibroblasts could have a higher energy turnover, shifting the balance of ATP to ADP. Regardless of the cause, depletion of ATP resources is catastrophic for any cell type, and a decrease in total ATP content reflects a decrease in the cell’s surplus capacity. Furthermore, as the energy consumption and production differ between cell types [20], and as the brain is the most energy demanding organ of the body with a metabolic activity notably constant over time [21,22], such an energy depletion is likely to have an even larger impact on neurons than on fibroblasts. As reduced energy capacity can sensitize cells during periods of increased energy demands it is likely that FOXG1 defective neurons are dysfunctional both during neurodevelopment and synaptic transmission (the most energy demanding function of the brain) [21]. This could in turn lead to neurodevelopmental disturbances and postnatal phenotypic manifestations of *FOXG1* syndrome.

In summary, our extensive assessment of mitochondrial function in FOXG1-fibroblasts suggests impaired mitochondrial homeostasis and thus provide novel insights into the pathogenesis of this syndrome. Our findings in fibroblasts should be expanded to neurons, preferably with the inclusion of a larger cohort, to establish the impact of mitochondrial homeostasis on impaired neurodevelopment and synaptic transmission that characterize *FOXG1* syndrome. If the loss of mitochondrial content can be confirmed in neurons, therapeutic strategies aimed at increasing mitochondrial biogenesis may be considered in future treatment strategies.

## Data Availability

The published article includes all datasets generated or analyzed during this study.

## Supplementary Materials

The following are available online at https://www.mdpi.com/article/10.3390/genes14020246/s1, Supplementary Table S1: Clinical features of the individuals investigated in this study and information on fibroblast cultures, Supplementary Table S2: RT-PCR and Sanger sequencing: Primers and Conditions, Supplementary Table S3: RT-qPCR: Taqman Probes, Supplementary Figure S1: Raw western blot membranes with indicated proteins.
