# Supplementary material for: Involvement of Mitochondrial Dysfunction in FOXG1 Syndrome
Source: Genes (Basel). 2023 Jan 17;14(2):246. doi: 10.3390/genes14020246 (PMC9957531; doi:10.3390/genes14020246)
Supplement: Supplementary file 1 [file genes-14-00246-s001.zip › genes-2139232-supplementary.pdf]

Supplementary Materials

Table S1. Clinical features of the individuals investigated in this study and information on fibroblast cultures.

Fibroblast	Coriell ID	Sex	Symptoms	*FOXG1* variant (NM_005249.5)	Age#	Passage
F1	GM27190	F	Global developmental delay (cannot sit unsupported, cannot walk or crawl), non-verbal, failure to thrive, microcephaly, epilepsy, hypotonia, dystonia, spasticity, cortical vision impairment, gastrointestinal reflux	c.924G>A p.(Trp308*)	6	8
F2	GM27246	F	Microcephaly; psychomotor retardation	c.256dup p.(Gln86Profs*35)	4	8
F3		F	Severe intellectual disability, microcephaly, refractory epilepsy, hypotonia, tetraplegia, abnormal MRI findings at age 45 years (bilateral hippocampal malrotation, supratentorial hypomyelination, simplified gyral pattern, atrophy of the cerebellum), cortical vision impairment, height -2SD	c.689G>A p.(Arg230His)	51	7
F4		M	Severe intellectual disability, microcephaly, epilepsy, hypotonia, wheelchair bound, abnormal MRI findings at age 4 years (delayed myelination), myopia, height -1SD	c.643T>G p.(Phe215Val)	9	7
F5		M	Intellectual disability, microcephaly, epilepsy, hypotonia, can walk very short distances, myopia, height -2SD	c.565C>T p.(Leu189Phe)	6	7
C1	GM00041	F	No symptoms	Normal	0	7
C2	GM00969	F	No symptoms	Normal	2	10
C3	GM08447	F	No symptoms	Normal	0	8
C4		F	No symptoms	Normal	35	7
C5	GM02036	F	No symptoms	Normal	11	10
C6	GM01652	F	No symptoms	Normal	11	10

#Age (years) when biopsy was taken; M, male; F, female; ID, Identification.

Table S2. RT-PCR and Sanger sequencing: Primers and Conditions.

Gene	Primer	Sequence
FOXG1	FW	5′-CATGTCGCCCTTCCTGTC-3′
	RV	5′-GAGTCAACACGGAGCTGTAG-3′
**PCR Master mix (**30 μl pr. reaction)		
FOXG1	5 μl cDNA	
	0.5 μl of each primer (20 μM)	
	0.5 μl dNTP mix (2.5 mM of each)	
	6 μl 5x Q-solution	
	3 μl 10x PCR buffer	
	0.1 μl HotStarTaq® DNA polymerase	
	14.4 μl sterile H2O	
**PCR conditions**		
95°C	15m	
95°C	1m	X40 cycles
52 °C	1m	
72°C	1m	
72°C	7m	
**Sequencing Master mix (pr. reaction)**		
FOXG1	2 μl PCR product	
	1 μl BigDye™ Terminator v3.1 Ready Reaction Mix	
	1.5 μl BigDye™ Terminator 5x Sequencing buffer	
	0.3 μl FW/RV primer	
	5.2 μl sterile H2O	
**Sequencing conditions**		
98°C	1m	
98°C	10s	X25 cycles

50°C	5s
60°C	4m

Table S3. RT-qPCR: Taqman Probes.

Target gene	Assay ID
TFAM	#Hs01082775_m1
PINK1	#Hs00260868_m1
PARK2	#Hs01038318_m1
DNML1	#Hs01552597_g1
MFN1	#Hs00966851_m1
MFN2	#Hs00208382_m1
OPA1	#Hs01047013_m1
GAPDH	#Hs02786624_g1
GUSB	#Hs00939627_m1

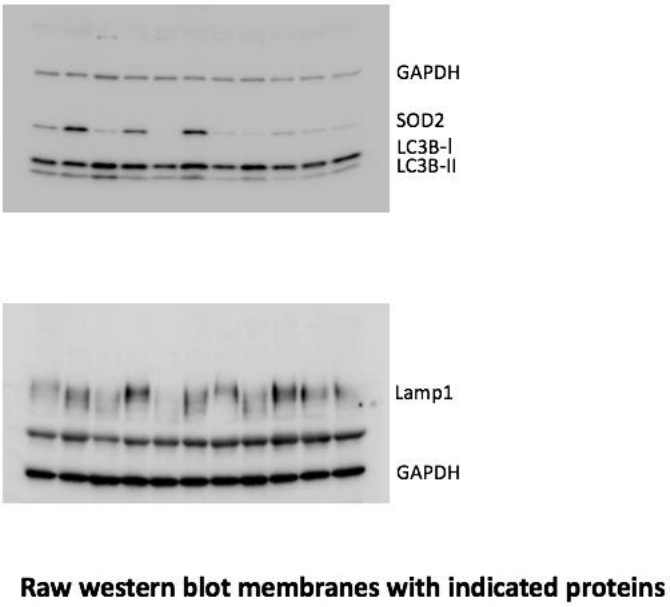

Figure S1. Raw Western blot membranes with indicated proteins.
